# An Overview of the Pathophysiology of Metabolic Changes and Their Sequence of Occurrence in Obese Diabetic Females: A Narrative Review

**DOI:** 10.7759/cureus.10947

**Published:** 2020-10-14

**Authors:** Azeezat A Oyewande, Beenish Iqbal, Lamis F Abdalla, Fazida Karim, Safeera Khan

**Affiliations:** 1 Family Medicine, California Institute of Behavioral Neurosciences & Psychology, Fairfield, USA; 2 Family Medicine, Lagos State Health Service Commission/Alimosho General Hospital, Lagos, NGA; 3 Pediatric Medicine, California Institute of Behavioral Neurosciences & Psychology, Fairfield, USA; 4 Internal Medicine, California Institute of Behavioral Neurosciences & Psychology, Fairfield, USA; 5 Psychology, California Institute of Behavioral Neurosciences & Psychology, Fairfield, USA; 6 Business & Management, Universiti Sultan Zainal Abidin, Terengganu, MYS

**Keywords:** diabetes mellitus, female diabetics, metabolic syndrome, metabolic changes and diabetes, adult females, obesity and diabetes

## Abstract

Obesity and diabetes both mediate their effects through insulin resistance and frequently co-exist. Insulin resistance is one of the key factors in the development of the metabolic syndrome. Adult females tend to develop obesity more frequently than males. One of the factors causing this difference is the pattern of changes that occur as females age from pre-menopausal to the post-menopausal stage, causing a change in the pattern of accumulation of fats. Several studies have explored and described the association between obesity and metabolic syndrome and their effect on type II diabetes.

We conducted our literature search using PubMed and Google Scholar as our primary databases. We selected a total of 49 articles for review after applying the inclusion and exclusion criteria and removing the duplicate articles. We chose the full-text articles that were published in the English language only. The selected studies were randomized controlled trials and review papers. The reviewed articles showed that visceral fat, central obesity, and fasting blood sugar of post-menopausal is higher than in pre-menopausal women and needs adequate management. More studies are needed in the future to explore the patterns of the metabolic changes in obese females to provide early and better management of diabetes and prevent related complications.

## Introduction and background

Diabetes mellitus may occur as a result of total damage to the islet β-cells in the pancreas, which is type I diabetes mellitus [1]. Type II diabetes mellitus (type II DM), however, occurs when the body has developed insulin resistance. The prevalence of type II DM varies from continent to continent, the Caribbean and North America top the list with a prevalence rate of one in eight people affected [2]. The increased risk of having diabetes is frequently linked to low, educational, socio-economic, occupational status [3]. Type II diabetes is a global health issue that has reached epidemic levels worldwide, affecting 425 million people and accounted for four million deaths in 2017 [4]. The 2019 global prevalence records around 463 million adults living with it [5]. Diabetes mellitus is a metabolic disorder caused by various ongoing metabolic changes and, in turn, alters multiple other metabolic functions. The endocrine system maintains metabolism and weight in the human body through hormones and growth factors. Hormones and growth factors like insulins, glucocorticoids, androgens, insulin and, thyroid hormones carry out their functions by modulating appetite, satiety, energy balance, and the contents and numbers of adipocytes. Therefore, there must be a balance between utilization and build-up of energy in the body [6,7].

Plasma glucose is also regulated by various organs and hormones in the body. The pancreas, for example, secretes insulin in response to high blood glucose, which is taken up by the tissues. This helps to mop up glucose when in excess. Pancreas also releases glucagon that acts on the liver to convert glycogen, other non-sugar precursors, to glucose when plasma glucose is low. The liver, in addition to regulating glucose, also helps with the metabolism of lipids through various hormone receptors. Thyroid hormones produced by thyroid glands also play a significant role in the metabolic activities in the body. When pro-inflammatory cells like cytokines produced by immune cells and adipocytes are released, they cause obesity. These are known to cause insulin resistance, which might then lead to type II diabetes [8]. As women grow from the middle-aged group towards menopausal age, several metabolic changes occur, affecting their lifestyle. An example is a reduction in the level of estrogens. These metabolic changes affect their activities, which leads to weight gain. Central fat distribution occurs along with other complications like dysfunctional lipid profile, dysglycemia, high blood pressure, diabetes mellitus, arthritis, sexual dysfunction, cancers, and other cardiovascular diseases [9].

The pattern of these metabolic changes has been studied in the general population, but it still needs to be explored in aging obese diabetic females. The sequence of these metabolic changes in adult diabetic females needs to be explored further. A knowledge gap still exists about the role of the environmental factors and the genetic predisposition of the body in developing central obesity, specifically in adult diabetic females. A further understanding and explanation of the common metabolic pathways in diabetes mellitus and metabolic syndrome are also needed to devise better management strategies.

In this review article, we aim to explore the patterns of metabolic changes that occur in aging diabetic females and the sequence of these events. We also aim to explore the role of central obesity in aging females and its effects on the development of the metabolic syndrome. This understanding will help devise better screening and management strategies in the aging female population.

## Review

We collected our data using PubMed and Google Scholar as our main databases. We also accessed some pathology textbooks for pathogenesis and definitions. The keywords used were metabolic changes, diabetes, adult females, metabolic changes both alone and in combination. MeSH keywords were also used to search for the relevant research papers. We collected various types of studies that were published in the past 20 years. We only selected the research papers that focussed on human research and specifically on obese adult females. Only full-text, peer-reviewed papers were chosen for the review. The numbers of papers extracted before and after using inclusion and exclusion criteria are shown in Table 1 below. A total of 49 articles were eventually selected after the application of inclusion and exclusion criteria. These range from systematic review, meta-analysis, and observational studies.

**Table 1:** The numbers of papers extracted before and after using inclusion and exclusion criteria.

Regular keywords used	Database used	Numbers of papers	After filters
metabolic changes	pubmed	1173622	384
diabetes	pubmed	746451	1081
adult female	pubmed	5442713	17584
metabolic changes and diabetes	pubmed	68711	91
obese female	pubmed	189625	597

MeSH KEYWORDS USED	DATABASE USED	NUMBERS OF PAPERS
metabolic changes	MeSH	384
female diabetics	MeSH	40680
metabolic changes and diabetics	MeSH	326799

An overview of diabetes mellitus as a metabolic syndrome

Diabetes mellitus is a group of metabolic disorders characterized by hyperglycemia, which results from defects in either insulin secretion or insulin action, or both. Long-term damage such as dysfunction, and failure of different organs, especially the eyes, kidneys, nerves, heart, and blood vessels, may arise as a result of chronic hyperglycemia of diabetes [10]. Certain organs like the liver, pancreas, and kidney along with their hormones help regulate plasma glucose levels in the body [8]. Type II DM accounts for 90-95% of those with diabetes mellitus, formerly known as non-insulin-dependent diabetes, type II diabetes, or adult-onset diabetes. The primary metabolic defect seen in type II DM is insulin resistance, which is the inability of the peripheral tissue to respond to insulin and Β-cell dysfunction that occurs as inadequate insulin secretion in the presence of insulin resistance and hyperglycemia [11]. Though, new discovery shows that the underlying defect in type II DM as evidenced in skeletal muscle tissue occurs as a result of disruption in the pathway of proteins metabolism and its transportation. And also, disruption during conversion of DNA to RNA. Future research in this disruptive pathway can help produce drugs that can halt its progress [12]. The prevalence of type II DM varies in different continents [2]. There is a projection that the number of people with type II DM will increase in the nearest future, especially in sub-Saharan African, due to change in lifestyle as a result of urbanization and the aging population. Women are considered to be more at risk for developing type II DM in sub-Saharan Africa, as African women have higher insulin resistance compared to Caucasians [13]. The incidence of type II DM has increased in the past years, as reported by some recent studies. It is expected to continue due to the predominant lifestyle, such as a sedentary lifestyle, intake of high processed foods, improved socio-economic status, and urbanization leading to decreased exercise [14]. Also, factors such as low socio-economic, low educational, and low occupational status have been linked as risk factors in type II DM, with about 425 million people developing it and accounting for four million deaths in 2017 [3,4]. However, there’s need for research as to why those with low socio-economic status living in high income society are more at risk of developing this condition.

Mechanisms linking obesity with diabetes

Obesity is defined as body mass index (BMI) > 30 kg/m^2^. It can further be subdivided into class I (BMI of 30 to <35), class II (BMI of 35 to <40), and class III (BMI of >40) [15]. Obesity is an imbalance between energy consumed and that expended, which is regulated by the neural and hormonal mechanism. It occurs when pro-inflammatory cells like cytokines produced by immune cells and adipocytes are released [8]. Once energy consumed exceeds energy expended, the excess calories are then stored in adipose tissue as triglycerides. The amount of stored energy (adipose tissue) is detected by the lipostat which then adequately regulate the quantity of food we eat and the amount of calorie we burn [11]. Obesity is a known worldwide epidemic that occurs as a result of a certain way of living, such as a sedentary lifestyle, intake of high canned food, improved socio-economic status, and urbanization [11]. There’s a notion that obesity is a show of affluence and good living. This probably might contribute to the increase in the number of those who are obese coming down with diabetes. In the United States, nearly two-thirds of women aged 40 to 59 years and about three-fourths of women 60 years and older are overweight body mass index (BMI), calculated as weight in kilograms divided by height in meters squared (>25 kg/m^2^). Furthermore, almost half of the women in these age groups are obese (BMI 30 kg/m^2^) [16]. Disruption in the sleeping pattern, deprivation of estrogen, and mood disorders have been linked to an increase in weight during aging [17]. Weight gain may also be due to physiologic changes of aging and a sedentary lifestyle. New research have linked loneliness as a prognosticator in the onset of type II DM as witnessed during the lockdown [18]. Around the fifth and sixth decade of life, a female gains about 0.7 kg every year irrespective of the previous body size or from where she originates [17]. Fats are distributed in different parts of the body, approximate measurements of fat accumulation in these sites can be done using the body mass index, which expresses weight in relation to height (kg per square meters). Also, body circumference which shows the ratio of the waist to hip circumferences and the measurement of the skin fold. This distribution of fat accumulation gives rise to some adverse effects, one of which is type II diabetes mellitus, especially the accumulation of fats in the trunk and abdominal cavity [19]. An example is the distribution of fat in the lower body in premenopausal women as against central distribution seen in those who are postmenopausal [17]. Central body fat (which is defined according to WHO classification as WC ≥ 102 cm and ≥ 88 cm for males and females, respectively, especially visceral fat, results in unfavorable metabolic effects, thereby increasing the risk of having diseases including type II DM [20]. However, some categories of people can be classified as being overweight or obese by virtue of their BMI. This does not necessarily translate to having metabolic abnormality. This brings in the role of genetic and environmental factors in the development of diabetes and obesity. However, a research has shown that those with higher-normal-weight BMI are at greater risk of developing metabolic syndrome [21]. Hence, the importance of considering the distribution of adipose fats in the body and way of calculation prior to classification, but without any metabolic abnormality. Therefore, obesity and diabetes have been linked as both mediate their effects through insulin resistance which is seen mostly in patients with type II DM and is an almost universal finding in obese DM people [22]. Diabesity is a term used due to an increase in the prevalence of diabetes and obesity worldwide as a result of poor eating habits and a sedentary lifestyle [23]. Impairment of Β-cell in type II DM shows the inefficiency of these cells to adjust themselves to the long-term demands of peripheral insulin resistance and increased insulin secretion [11]. Most African women have more insulin resistance than Caucasian women. A study done in Sub-Saharan Africa (SSA) shows that the burden of the risk factors for type II DM, especially obesity, is higher in women than men. The differences are due to factors like improved lifestyle and urbanization with advanced age. Even though the prevalence of diabetes does not differ by gender, but death attributed to type II DM in SSA is higher in females, which may be due to differences in beliefs and access to care [13]. Insulin resistance causes a reduction in uptake of glucose in muscle and adipose tissues and this leads to suppression of hepatic gluconeogenesis [11]. The effects of obesity include but are not limited to exaggerated symptoms of hot flushes and sexual dysfunction in midlife women [24]. The quantity of the adipose tissue stored is sensed by the lipostat, which regulates food intake and energy expenditure [11]. To regulate this, the afferent system generates humoral signals from adipose tissue (Leptin), pancreas (Insulin), and stomach (Ghrelin). A central processing unit, in the hypothalamus, then integrates the affected signals. The effector system then executes its function from the hypothalamic nuclei in the form of feeding behavior and energy expenditure. Leptin plays a principal role in energy homeostasis as it is present and enters the brain in a concentration proportionate to body fat mass, suppressing the intake of food [25].

Pathophysiology of metabolic syndrome and the role of central obesity in the development of insulin resistance

**Figure 1:**
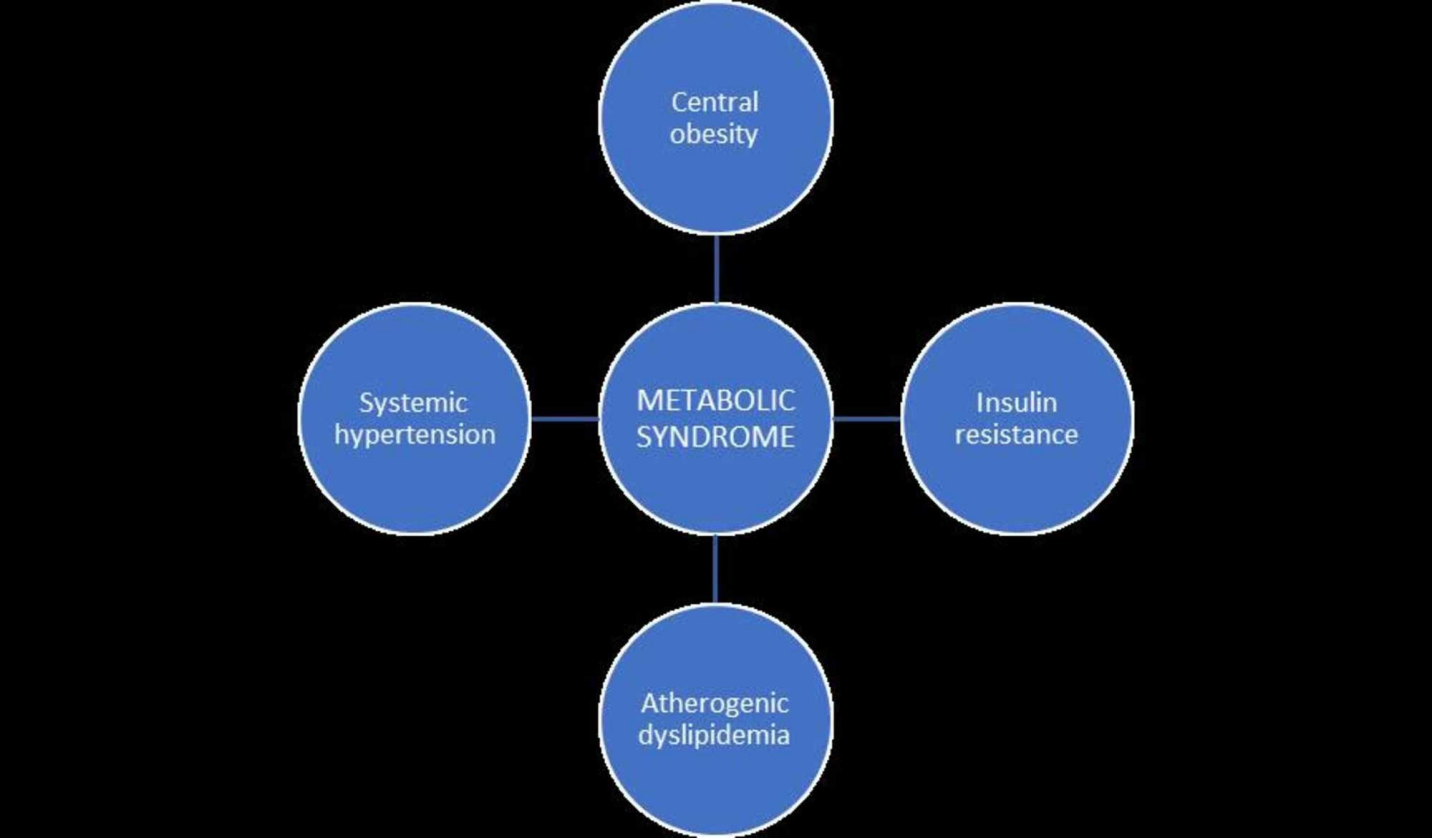
The various components of metabolic syndrome.

Metabolic syndrome (MetS) is a collection of cardiovascular risk factors that are characterized by obesity, central obesity, insulin resistance, atherogenic dyslipidemia, and hypertension, which has been linked to insulin resistance as a key factor in the development of the metabolic syndrome [26,27]. Of the general population, about 17-25% have MetS, out of which 59 to 61% occur in people with DM [28,29]. Metabolic syndrome is the complication that arises from obesity. This comprises of insulin resistance, type II DM, dyslipidemia, hypertension, abdominal obesity, hepatic steatosis, sleep apnea, and lipid accumulation [30]. Risk factors for MetS can be either modifiable or non-modifiable factors. Modifiable factors include physical inactivity/sedentary lifestyle, urbanization, smoking, alcohol consumption, family income, and level of education. While some of the non-modifiable factors are increasing age, female gender, and family history of diabetes [31]. Age is an important risk factor and plays a significant role in the prevalence of MetS all over the world [32]. One of the researches shows that the higher the age, the increase in the prevalence of MetS. As observed in a Finnish study, which shows an increase in the prevalence of MetS with increasing age in women [33]. There is an increase in the prevalence of MetS at an exponential rate globally [34], which is due to an increased prevalence of type II DM, hypertension, obesity, and other cardiovascular diseases [35]. In the adult population, it is estimated that the prevalence is 20-25% [36]. Most people with type II DM or impaired glucose tolerance have metabolic syndrome [29]. Diabetes mellitus, as already observed, increases the likelihood of developing metabolic syndrome [37]. In a longitudinal cohort study done to inquire into the relationship of changes in MetS and its components with the risk of type II DM in South Korea, it was discovered that people with prolonged changes in MetS components developed type II DM. This shows that metabolic syndrome abnormality can predict the onset of type II DM [38]. Visceral fat plays a vital role in the development of MetS [39]. Symptoms of MetS occurring due to abdominal obesity mostly occur in those with normal waist circumference and body mass index [14]. There are different diagnostic criteria for the MetS. According to the Japan Committee of the Criteria for MetS, they consider visceral fat (waist circumference) as a major factor plus at least two abnormal glucose metabolism, abnormal lipid metabolism, and high blood pressure [40,41]. Some studies have shown that MetS in Asian people have a lesser link with risk of becoming diabetic [42]. The criterion for the MetS includes five variables, namely, abdominal obesity, increased triglycerides, low high-density lipoprotein (HDL), elevated blood pressure, and history of diabetes mellitus or impaired fasting glucose state [43]. They are shown in Figure 1 below.

**Figure 2:**
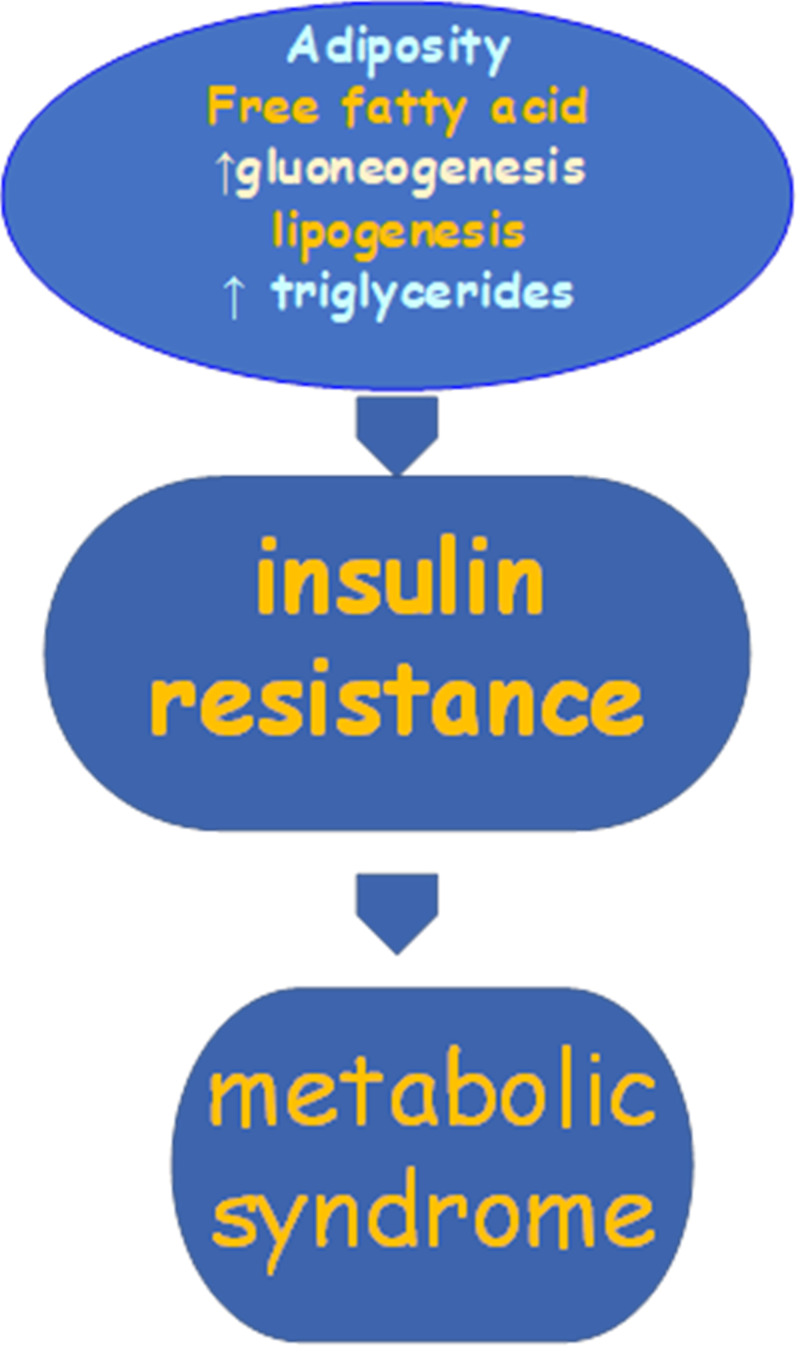
Pathophysiology of metabolic syndrome.

The common risk factors of MetS include obesity, aging, sedentary lifestyle, diabetes mellitus, coronary heart disease, and lipodystrophy [44]. Also, genetic tendency is a factor in MetS with some studies stating the involvement of HDL genes in about 70%, with different prevalence among ethnic groups [45]. Socio-economic status, age, lifestyle changes also play a major role in the pathogenesis [34]. Central obesity is filled with active brown adipocytes, which is metabolically active and has been confirmed to have a strong link with insulin resistance, dyslipidemia, hypertension, and atherosclerotic heart disease than obesity. Its particular role in a patient with metabolic syndrome can't be explained [46]. Obesity, particularly in the presence of increased visceral fat, raises the risk of several adverse metabolic health consequences, including dysglycemia or frank type II diabetes mellitus, dyslipidemia, and hypertension. Figure 2 below shows the pathophysiology of metabolic syndrome.

There is a different pattern of presentation of a component of the metabolic syndrome. An example is a study done in Seychelles, where high blood pressure and adiposity occurred mostly among the components. However, in another study, reduced HDL-cholesterol, which was noticed to be significantly lower in males than females, and central obesity, which is seen to occur first before others were prominent components of metabolic syndrome among those examined [37]. In terms of the pattern of abnormality of the lipid component of MetS, women had higher low-density lipoprotein-cholesterol (LDL-C), total cholesterol (TCHOL), and high-density lipoprotein (HDL-C) but a lower triglyceride when compared to the men with MetS [37]. Another study discovered that LDL-C was the most common lipid abnormality in patients with MetS studied [47]. In terms of hypertension, a study found females having an incidence remarkably higher than men with MetS. A similar finding was seen in the Middle East and Nigeria [28,37]. A study on prevalence and gender distribution of the metabolic syndrome reported the presence of all components of MetS in a small proportion of the subjects, unlike the report gotten in another study where some components of MetS were not present in patients with type II DM. Another study done among the female population in a sub-Saharan African setting shows central obesity as the most prevalent component of metabolic syndrome with variation among those living in a rural and urban setting [48]. Several co-morbidities such as pro-thrombotic and pro-inflammatory states, non-alcoholic steatohepatitis, and reproductive disorders and malignancy have been associated with metabolic syndrome [49]. Aging results in a decrease in lean body mass, which decreases the resting metabolic rate. It can result in a decrease in both basal and total energy expenditure, and unless a woman adjusts her caloric intake and consciously increases her physical activity (PA) level, a state of positive energy balance results, with associated weight gain [9]. Health talk during clinics on avoiding a sedentary lifestyle through a healthy diet and regular physical exercise that will prevent central obesity at least 30-minutes daily should be emphasized during clinic visits to female patients. MetS increases the risk of type II diabetes, cardiovascular disease, and all-cause mortality [34].

**Figure 3:**
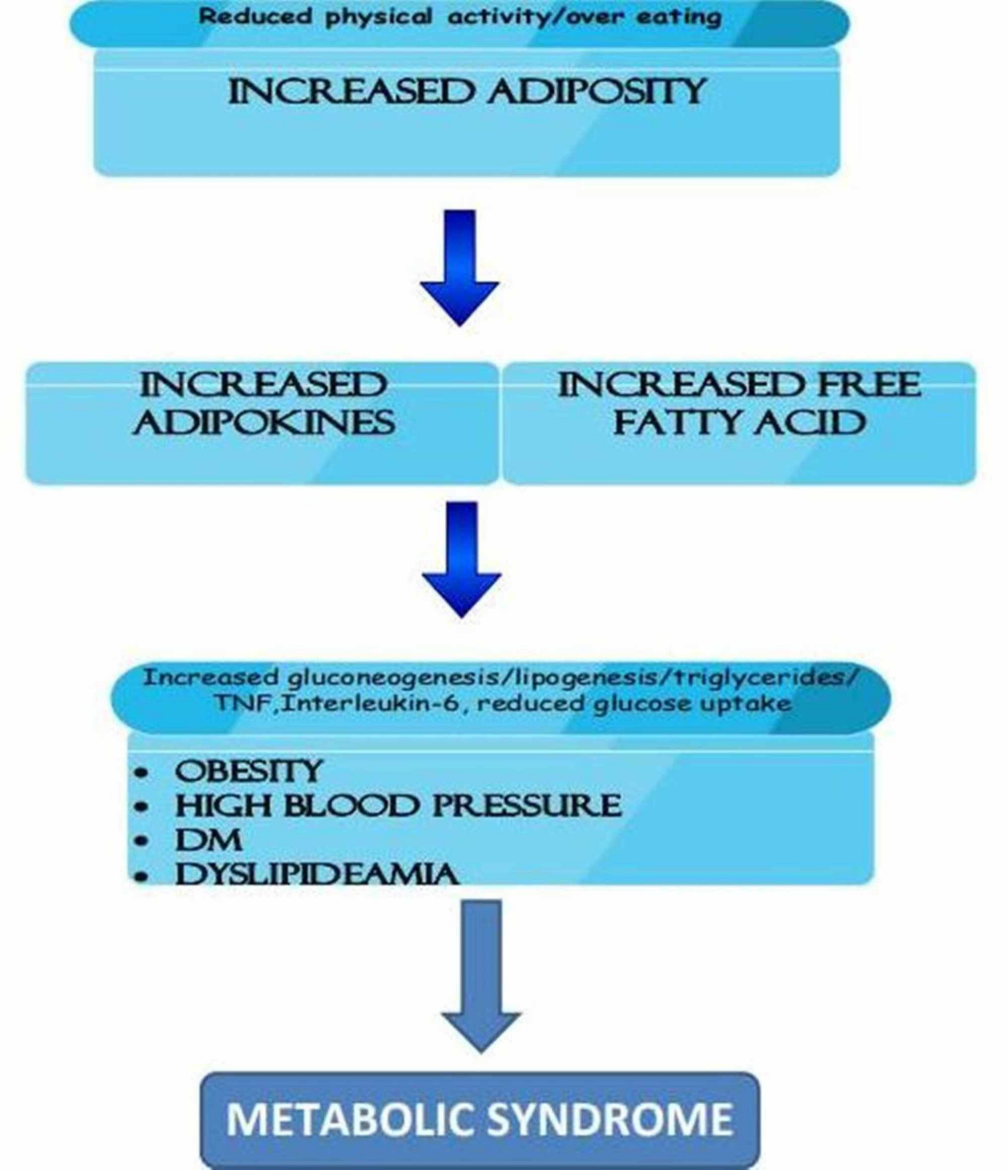
Link between obesity, diabetes mellitus and metabolic syndrome.

The pathophysiology occurring in metabolic syndrome and the link between obesity, diabetes mellitus, and metabolic syndrome are shown in Figure 3 below.

Some of the reviewed studies showing the effect of obesity on metabolic syndrome are shown in Table 2 below.

**Table 2:** Some of the reviewed studies on effects of obesity on metabolic syndrome. Type II DM: Type II diabetes mellitus; HDL-C: High-density lipoprotein-C.

Author/year of publication	Type of study	Purpose of the study	Results/conclusion
Agardh et al./2011 [3]	Systemic and meta-analysis	To determine the relationship between the incidence of type II DM and socioeconomic position	The low socio-economic position was associated with the risk of coming down with type II DM in lower-income and high-middle and countries and overall
Goedecke et al./2017 [13]	Clinical review	To determine the prevalence of type II DM among women in sub-Saharan Africa (SSA)	Women in SSA are more insulin resistant than the Caucasian and have been projected to have the highest rate of Type II DM.
Abdulnour et al./2012 [20]	Observational longitudinal study	To find out the transformation that occurs in the body composition and cardiometabolic profile when transitioning menopausal stage	Visceral fat, fasting blood sugar, central fat mass, and HDL-C (0.05>p<0.01) were all noticed to increase markedly in peri and post-menopausal women after three years of the study.
Ogbera /2010 [37]	Observational studies	Determining the prevalence of metabolic syndrome and to note the gender characteristics similarity among patients with Type II DM	There’s a high prevalence of Mets in Type II DM in both genders and central obesity was the commonest occurring defining indices while the least is elevated triglyceride levels
Ford et al./2002 [45]	Report	Finding how common metabolic syndrome is among adults in the US	Prevalence increases with aging with a peak of around 20-29 years. African Americans had a 57% prevalence higher than men likewise among Mexican Americans with the women having a 26% higher prevalence than men.
Fezeu et al./2007 [48]	Observational study	To know how common metabolic syndrome is and find the link between components of the metabolic syndrome and central obesity and HOMA insulin resistance index.	Increase in prevalence of metabolic syndrome in both gender in those living in an urban area but none in the rural area. None of the subjects had all four components. Most had two combinations of three components.

Limitations

Most of the studies that were reviewed did not point out in detail, the sequence of occurrence of each component, and if there's a regular sequence that the components of the metabolic syndrome do occur. If research is done in this aspect, most females with diabetes will be adequately monitored and managed, and complications from this will be greatly reduced.

## Conclusions

The ability to predict the sequence of occurrence of metabolic changes in an adult obese female diabetic is the key to identify which female diabetics are prone to develop central obesity and metabolic syndrome. These metabolic changes are due to change in pattern of fat distribution from peripheral to central pattern, predisposing them to become obese. This can trigger development of other components of metabolic syndrome. A special classification and algorithm can be considered in female diabetic patients approaching menopause. Randomized controlled trials focusing on predicting the sequence of metabolic abnormality can guide relative risk prediction and clinical management decisions.
